# Comparative single-cell analysis of the adult heart and coronary vasculature

**DOI:** 10.1007/s00335-022-09968-7

**Published:** 2022-11-19

**Authors:** Saranya Balachandran, Jelena Pozojevic, Varun K. A. Sreenivasan, Malte Spielmann

**Affiliations:** 1 Institute of Human Genetics, University Hospital Schleswig-Holstein, University of Lübeck and Kiel University, Lübeck and Kiel, Germany; 2 Human Molecular Genetics Group, Max Planck Institute for Molecular Genetics, Berlin, Germany; 3 DZHK e.V. (German Center for Cardiovascular Research), Partner Site Hamburg, Kiel, Lübeck, Germany

## Abstract

The structure and function of the circulatory system, including the heart, have undergone substantial changes with the vertebrate evolution. Although the basic function of the heart is to pump blood through the body, its size, shape, speed, regeneration capacity, etc. vary considerably across species. Here, we address the differences among vertebrate hearts using a single-cell transcriptomics approach. Published datasets of macaque (*Macaca fascicularis*), mouse, and zebrafish hearts were integrated and compared to the human heart as a reference. While the three mammalian hearts integrated well, the zebrafish heart showed very little overlap with the other species. Our analysis revealed a mouse-specific cell subpopulation of ventricular cardiomyocytes (CM), represented by strikingly different expression patterns of specific genes related to high-energy metabolism. Interestingly, the observed differences between mouse and human CM coincided with actual biological differences between the two species. Smooth muscle and endothelial cells (EC) exhibited species-specific differences in clustering and gene expression, respectively, which we attribute to the tissues selected for sequencing, given different focuses of the original studies. Finally, we compared human and zebrafish heart-specific fibroblasts (FB) and identified a distinctively high expression of genes associated with heart regeneration following injury in zebrafish. Together, our results show that integration of numerous datasets of different species and different sequencing technologies is feasible and that this approach can identify species-specific differences and similarities in the heart.

**Supplementary Information:**

The online version contains supplementary material available at 10.1007/s00335-022-09968-7.

## Introduction

The heart is a complex organ at the center of the circulatory system, which pumps blood through the body, enabling the exchange of nutrients, respiratory gasses, metabolic waste, etc. It has evolved over millions of years, from simple structures like those seen in insects and worms to powerful four-chambered mammalian hearts. In vertebrates, a multi-chambered heart exists together with a closed vascular system composed of arteries, veins, and capillaries. The simplest vertebrate heart belongs to fish and consists of two chambers, while most reptiles (except for crocodiles and alligators) have a three-chambered heart, consisting of two atria and a ventricle (Stephenson et al. 2017). Finally, mammals and birds have a four-chambered heart that consists of two atria and two ventricles, where the right ventricle pumps deoxygenated blood to the lungs, while the left ventricle pumps blood rich in oxygen to the rest of the body. The four chambers of the heart are attached to major veins and arteries that bring blood into (e.g., vena cava) or carry blood away (e.g., aorta) from the heart, while the coronary arteries (coming out of the aorta) supply blood to the heart muscle itself. The heart is composed of multiple cell types, including cardiomyocytes (CM) that generate contractile forces, smooth muscle cells (SMC) and pericytes (PC) that form blood vessels and play key roles in vascular contraction, tone, and integrity, endothelial cells (EC) that regulate exchange between the bloodstream and the surrounding tissue, fibroblasts (FB) that produce connective tissue and other cell types such as neuronal-, lymphoid-, myeloid cells and adipocytes (Alberts et al. 2002; Litviňuková et al. 2020).

Single-cell sequencing technologies (sc-seq) have enabled the detailed characterization of these cell types based on their gene expression profiles (Sreenivasan et al. 2022). The technology has facilitated the understanding of development, differentiation, homeostasis and diseases at cellular resolution (Smajić et al. 2022; Huang et al. 2022). Within the last few years, breakthrough sc-seq methods have been applied to analyze the cellular composition of various organisms and more specifically organs, including the heart (Cao et al. 2020). Moreover, the Human Cell Atlas initiative was established with the aim to map the entire human body in adults and in embryonic stages (https://www.humancellatlas.org) (Cao et al. 2019, 2020).

With so many rich datasets spanning even evolutionarily distant species available at our fingertips, here we set out to integrate the sc-seq data from the hearts of adult mouse, crab-eating monkey (*M. fascicularis*), and human, as well as the recently published zebrafish (*Danio rerio*) adult heart dataset (Vidal et al. 2019; Zhang et al. 2020; Litviňuková et al. 2020). The data from the two-chambered zebrafish heart was of particular interest, since it possesses the ability to regenerate upon injury, which was recently attributed to the transient cell states with fibroblast-like characteristics (Hu et al. 2022). In contrast, mammalian hearts cannot regenerate after an injury, instead leaving scar tissue with decreased functionality.

## Methods

Single-cell feature barcode matrices of heart tissue were obtained from ERP123138 for adult humans (Litviňuková et al. 2020), E-MTAB-7869 for adult mice (Vidal et al. 2019) and GSE117715 for adult macaques (Zhang et al. 2020). Orthologous genes from the BioMart (Ensembl Genes 107) were used to create the feature barcode matrices of the three species. Individual species were processed separately prior to integration. Cells with more than 1500 UMIs and 1000 genes and less than 10% of mitochondrial genes and 10% of ribosomal gene counts were used. Genes detected in more than three barcodes were retained.

Seurat v4 was used for the downstream analysis. First, each species was log normalized using the standard workflow (as described in the Seurat—Guided Clustering Tutorial) with 2500 highly variable features. Principal component analysis (PCA) was done on the normalized and scaled expression matrix. For integration, the common features between the datasets were found using the Select Integration Features function in Seurat, which was then used to identify the anchors based on the reciprocal PCA method, where the human dataset was used as the reference. The datasets were integrated with Seurat due to its enhanced performance with huge data (Tran et al. 2020). The integrated data was then scaled and PCA was performed. The nearest neighbor graph was built with 30 PCs, which was then clustered using the Seurat *FindClusters* function based on the Louvain algorithm. The Wilcox algorithm of the Seurat *FindAllMarkers* function was used to identify the differentially expressed genes in each of the clusters. The gene markers provided in the three studies were compared to those identified by us to annotate the major clusters.

For sub-clustering, the raw count data for endothelial, smooth muscle cells, PC, ventricular CM, atrial CM, and fibroblast cell types were individually subset and 1500 highly variable features were used for principal component analysis. These subsets of cells were integrated across the species based on Harmony at the reduced PCA space. Due to the efficiency of harmony in integrating identical cell types sequenced by different technology, it was a method of choice for integrating the cellular subpopulation (Tran et al. 2020). The nearest neighbor graphs were built on the harmony-based reduction. The Louvain clustering approach was performed based on the top 10 reduction components of the integrated datasets. The Wilcox algorithm of the Seurat *FindAllMarkers* function was used to identify the differentially expressed genes in each of the clusters. The cell type markers from Litviňuková et al. 2020 were used for identifying the cellular sub-clusters.

For zebrafish heart tissue, the single-cell-barcode matrix was downloaded from GSE159032 (Hu et al. 2022). The orthologous genes in humans to the gene identifiers of zebrafish were compiled. The cell-barcode matrix of human and zebrafish were subset to contain only the orthologous genes. The raw count data for FB were obtained from both human and zebrafish datasets. The datasets were then log normalized and scaled before merging. The merged dataset was integrated using Harmony, correcting for the species and samples. We then built a hierarchical cluster tree based on harmony reduction using the BuildClusterTree function in Seurat. The genes associated with the regeneration of FB in the zebrafish dataset were visualized in the integrated data to quantify the differential expression in humans and zebrafish.

Gene ontology analysis was performed for the ventricular CM cluster that was specific to mice using g: Profiler web server (Raudvere et al. 2019). The top 500 markers with adjusted *p*-value < 0.05 and a higher percentage of cells expressing the gene in the cluster were provided as input. The gene ontological biological process terms that had a negative log adjusted *p*-value > 10 were plotted.

## Results

**Fig. 1:**
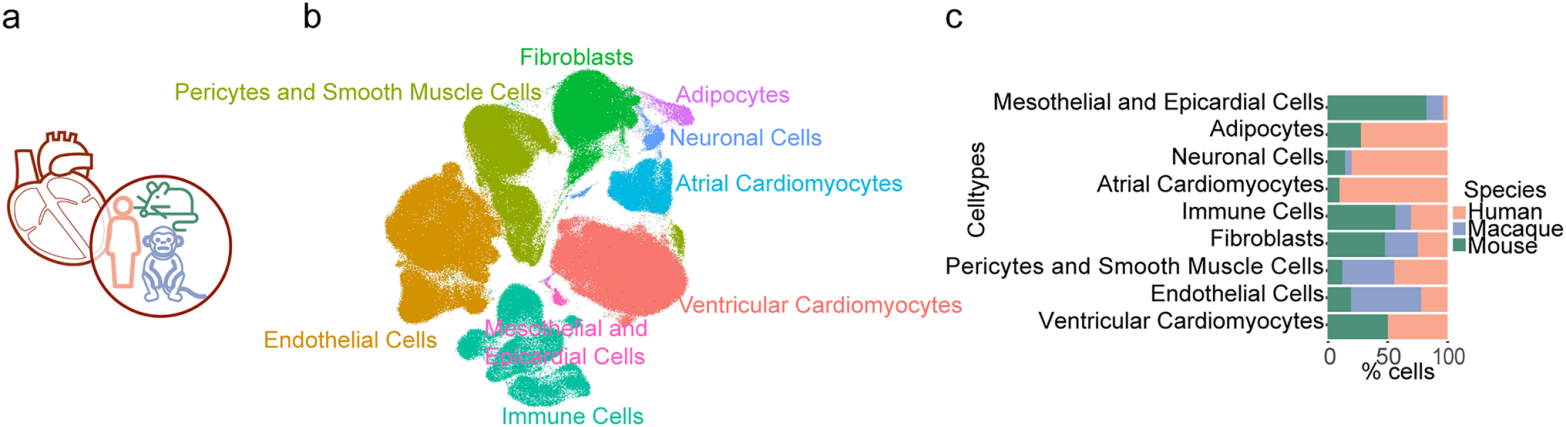
Cell types and composition of adult mammalian hearts. **a** Integration of single-cell heart transcriptome data from human, macaque, and mouse. **b** 2D UMAP embedding of the major cell types after integration. **c** Percentage contribution of each species to the cells in the major cell types

The adult hearts from the three species, namely human, macaque (*M. fascicularis*) and mouse have been characterized using single-cell RNA sequencing (Vidal et al. 2019; Litviňuková et al. 2020; Zhang et al. 2020). Human and mouse datasets were sequenced by Chromium Single-Cell 3′ protocol (10 × Genomics) and macaque by STRT-seq. The adult human heart and mouse heart datasets consist of 451,513 cells and 12,710 cells, respectively, whereas the macaque heart dataset generated only from the aorta and coronary arteries consists of 7989 cells. To account for the variation in sequencing technologies and the cell types sequenced, we integrated the three species with Seurat’s reciprocal PCA-based method followed by clustering (Fig. 1a, Methods). The marker genes provided in the published datasets were used to annotate the clusters (Supp Fig. 1a). We were able to identify the 9 major cell types composing the adult mammalian heart (Fig. 1b). Ventricular CM, atrial CM and adipocytes consisted of only human and mouse tissues due to the sequencing focus of the macaque heart tissue (Fig. 1c, Supp Fig. 1b). Endothelial cells, PC, and SMC had a higher percentage of cells originating from the macaque tissue. 50% of the FB were composed of mouse tissue and the rest were equally represented by humans and macaques.

We then investigated the cell subpopulations of atrial and ventricular CM, endothelial cells, PC and SMC and FB. We compared the identified cell subtypes with the adult human heart study (Litviňuková et al. 2020) and saw that FB, atrial CM and EC were in agreement with the cell subpopulations of the human heart atlas, though the approach to integrate and sub-cluster the cell types was not identical to our study (Supp Fig. 2). Although the cells integrated well, we observed the segregation of macaque cells in the FB3 cluster (Supp Fig. 2b).

**Fig. 2:**
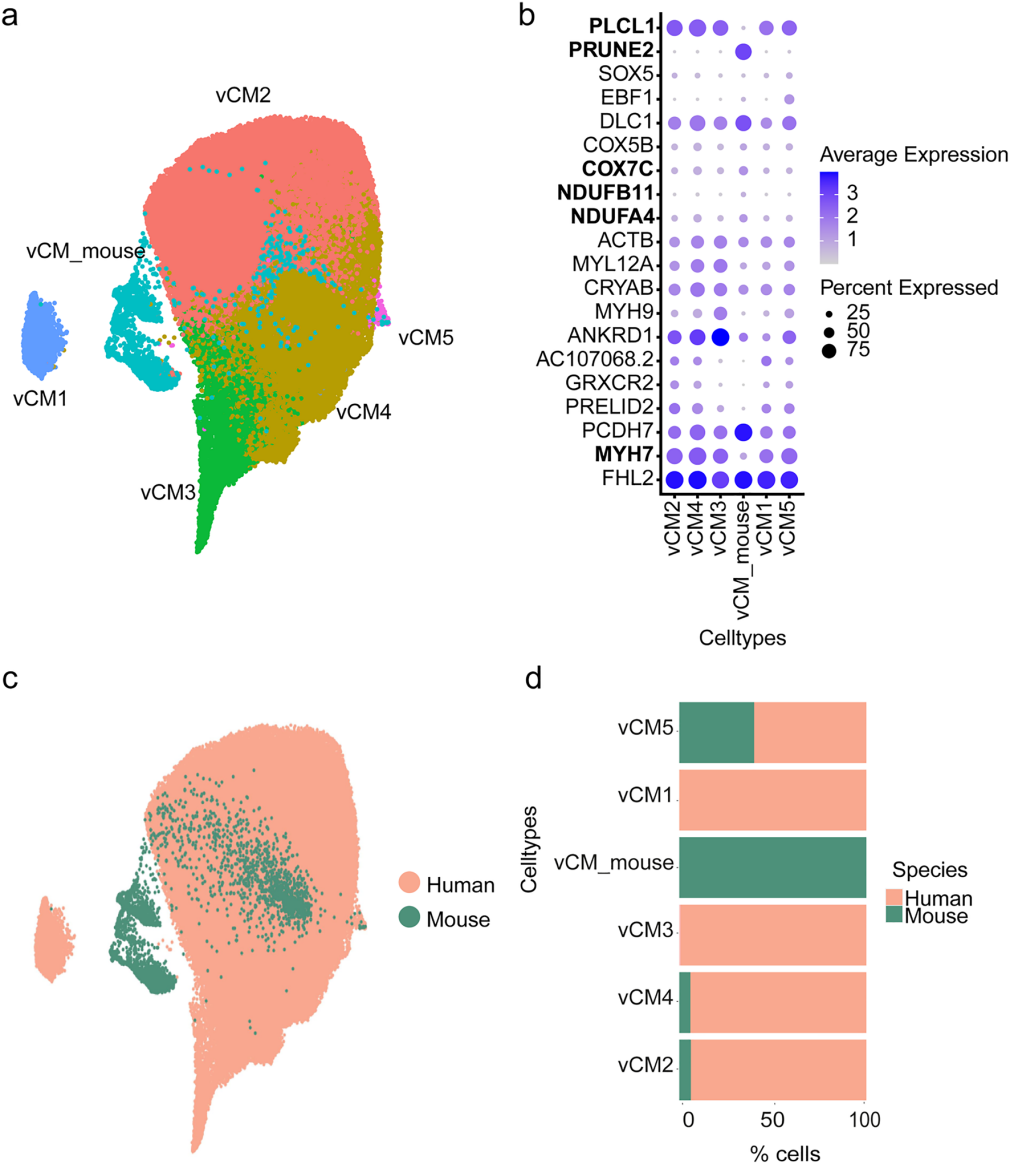
Ventricular CM. **a** 2D UMAP embedding of the cell subpopulations of ventricular CM after integrating the cells from the two species. **b** Dot plot of the cell type-specific marker genes. The key genes showing differential expression between species are written in bold. **c** 2D UMAP embedding of the integration of the cells from the two species. **d** Contribution of each species to the percentage of cells in the ventricular CM

On sub-clustering of the ventricular CM, we identified 6 cell subtypes (Fig. 2a). By comparing the cell type compositions, we noticed a cluster consisting exclusively of mouse cells that did not integrate into the human ventricular CM (Fig. 2c, d). We sought to analyze the marker genes of this mouse-specific cell type and saw that *Myh7* and *Plcl1* had a lower expression compared to other clusters, while *Prune2* had a higher expression comparatively. On the other hand, *Ndufa4, Ndufb11 and Cox7c*, marker genes of human vCM4 that encode mitochondrial respiratory chain enzymes, showed a slightly higher expression in mice than in any human cell types (Fig. 2b, Supp Fig. 3).

**Fig. 3:**
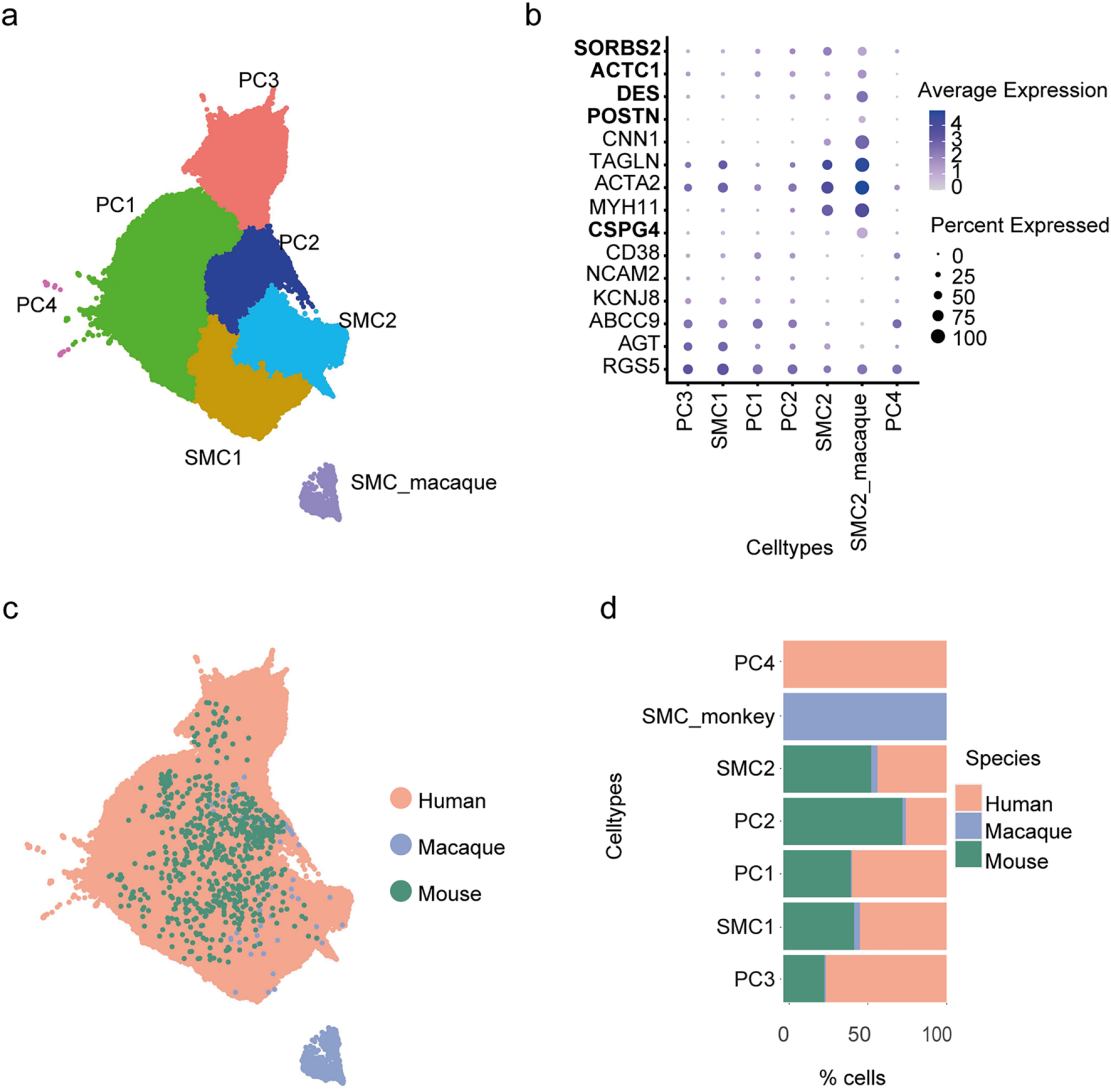
Pericytes and smooth muscle cells. **a** 2D UMAP embedding of the cell subpopulations of PC and SMC after integrating the cells from the two species. **b** Dot plot of the cell type-specific marker genes. **c** 2D UMAP embedding of the integration of the cells from the three species. **d** Contribution of each species to the percentage of cells in the PC and smooth muscle cells

We identified 7 subclusters of the PC and SMC (Fig. 3a). Five of these cell types consisted of human and mouse samples (Fig. 3c, d). PC4 had only cells from the human dataset but it could represent unknown cell states or doublets, as stated by the authors of the study (Litviňuková et al. 2020). Interestingly, we also saw a new smooth muscle cell type SMC_macaque (Fig. 3c, d) which expressed the SMC markers of humans such as *MYH11, ACTA2, TAGLN* and *CNN1* as well as other marker genes such as *POSTN*, *DES, ACTC1, SORBS2* which were previously described as aortic and coronary artery-specific (Zhang et al. 2020) (Fig. 3b). In addition, they expressed *CSPG4*, a marker gene for PC (Fig. 3b).

**Fig. 4:**
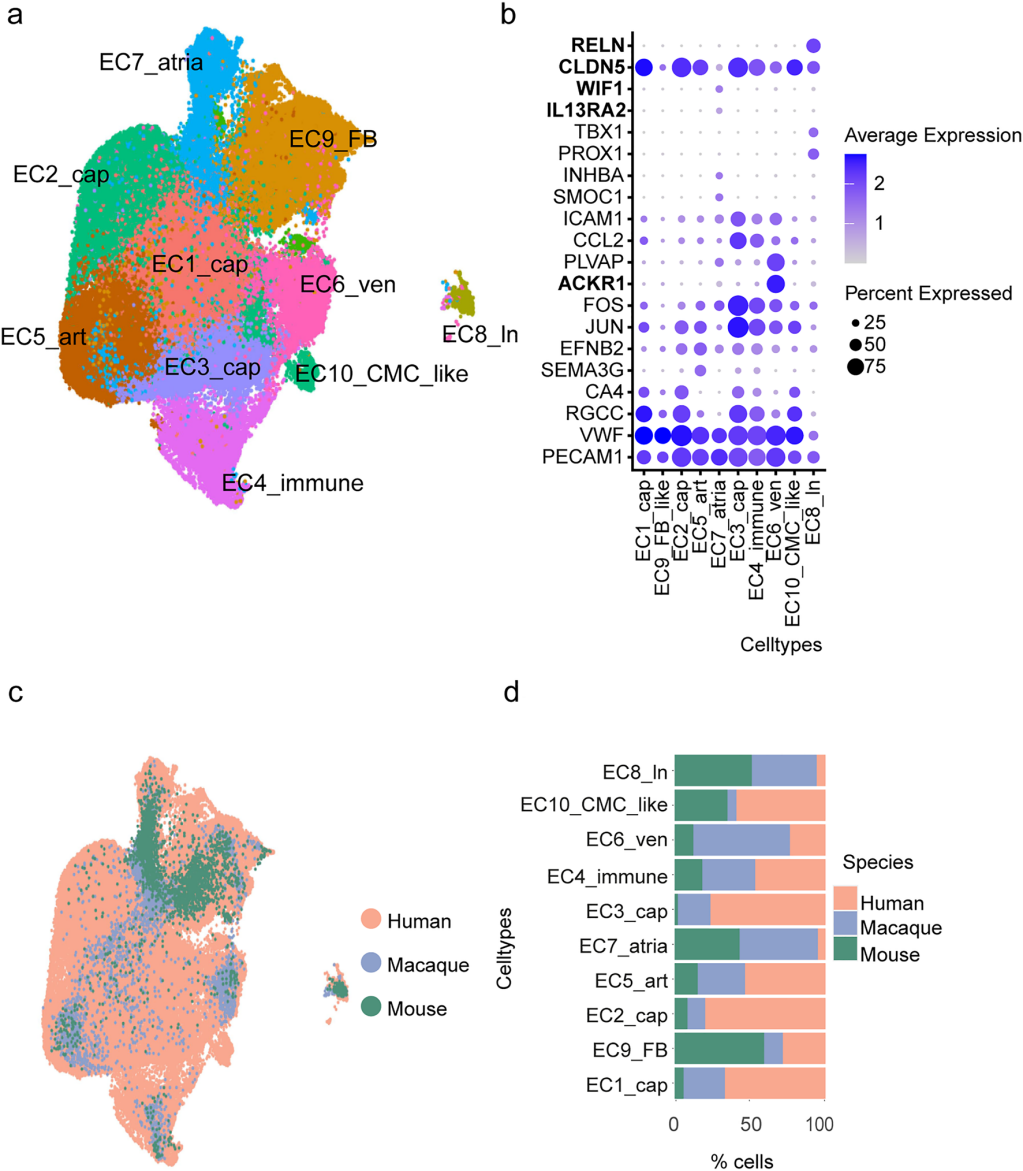
Endothelial cells. **a** 2D UMAP embedding of the cell subpopulations of EC after integrating the cells from the 2 species. **b** Dot plot of the cell type-specific marker genes. EC_cap—capillary, EC_FB—fibroblast, EC_art—arterial, EC_atria—atrial, EC_ven—venous, EC_CMC_like—cardiomyocyte_like, EC_ln—lymphatic. **c** 2D UMAP embedding of the integration of the cells from the three species. **d** Contribution of each species to the percentage of cells in the endothelial cells

Next, we investigated the EC and could show that they were composed of 10 cell subpopulations (Fig. 4a), defined by the marker genes as described by human heart atlas study (Litviňuková et al. 2020). The cells of the three species integrated well and we did not observe any species-specific clusters. However, EC7_atria and EC8_ln were mainly composed of macaque and mouse cells (Fig. 4c, d). Additional genes highly expressed in the EC7_atria cluster include *IL13RA2* and *WIF1*, associated with aortic artery EC in macaque (Zhang et al. 2020). In contrast, lymphatic EC8_ln cells showed high expression of *RELN*, a gene expressed in macaque lymphatic endothelial cells, consistent with the cell type specificity in both macaque and human. Furthermore, we observed that *CLDN5*, previously associated with coronary artery-specific EC in macaques, was expressed in all clusters (Fig. 4b).

Finally, we wanted to study the differences between human and zebrafish hearts. Humans and zebrafish are evolutionarily distant, resulting in only 500 orthologous genes, thus making it challenging to integrate the datasets compared to integration of the mammalian species (Qiu et al. 2022). The low heterogeneity in integrating an organ datasets versus the whole organism datasets adds another level of complexity due to the low number of integration anchor points (Lähnemann et al. 2020; Argelaguet et al. 2021).

**Fig. 5:**
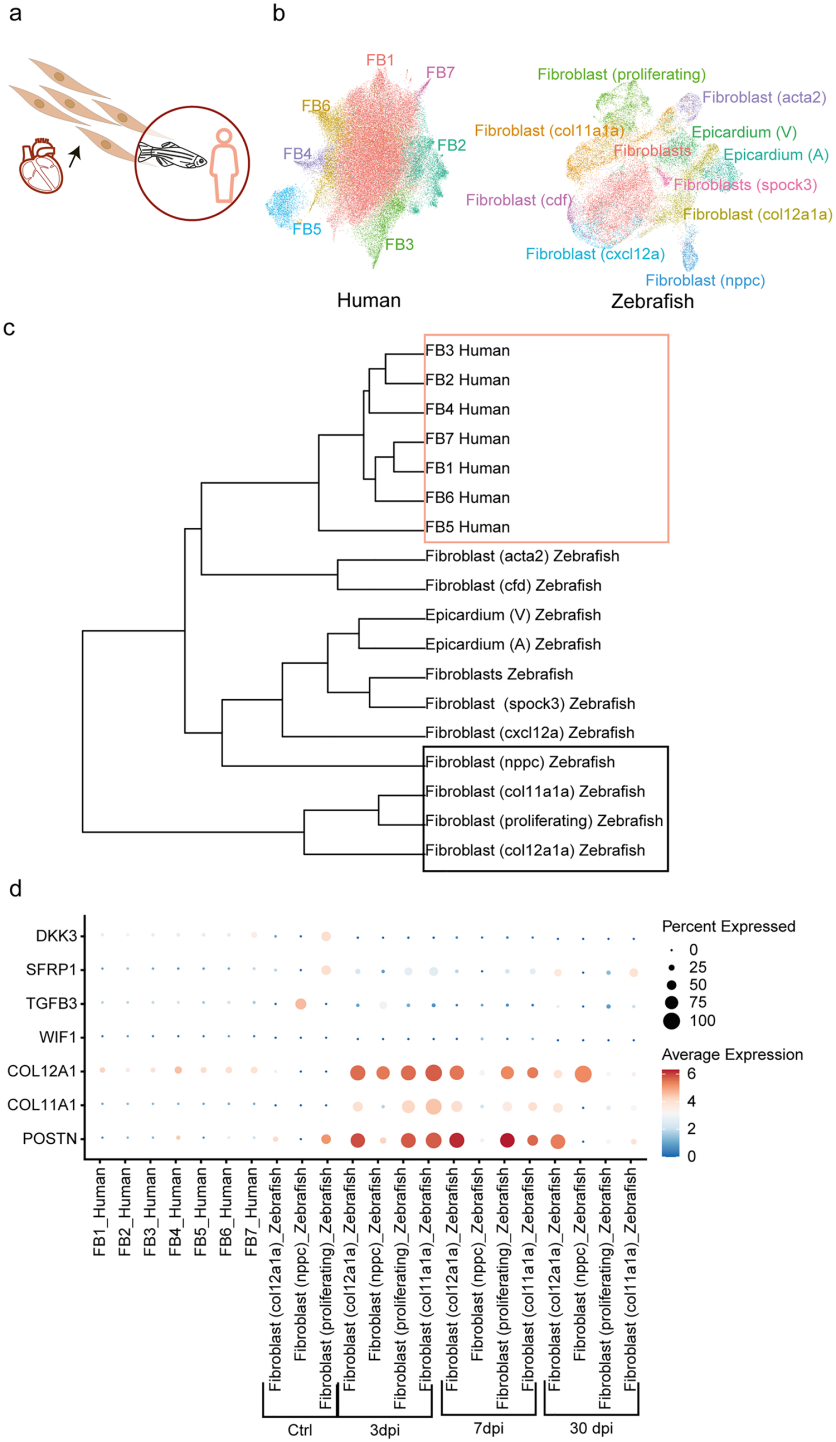
Integration of human and zebrafish cardiac FB. **a** Single-cell integration of FB of heart datasets from human and zebrafish. **b** UMAP embedding of the integration of FB from human and zebrafish. **c** Hierarchical cluster tree analysis of the fibroblast subpopulations. The orange box encircles the populations of the human FB, while the black box encircles the key cell states involved in the regeneration of FB post-injury in zebrafish. **d** Dotplot representation of the expression of genes essential for regeneration of FB. The expression in zebrafish is split by control and number of days post-injury (dpi)

Therefore, we focused on the integrating cardiac FB of zebrafish and human datasets, since zebrafish FB have recently been shown to play a major role in cardiac regeneration (Fig. 5a, Supp Fig.4a) (Hu et al. 2022). The zebrafish FB consisted of 11 cell subpopulations, whereas the human FB had 7 subpopulations (Fig. 5b). The regenerating FB have been shown to form a new cell state 3 days post-injury, which consists of fibroblast (proliferating), fibroblast (*nppc*), fibroblast (*col11a1a*) and fibroblast (*col12a1a*) (Hu et al. 2022) (Supp Fig. 4b). On hierarchical cluster tree analysis after integration, the cell subpopulations from human datasets were distant from the 4 zebrafish-specific cell states known for regeneration (Fig. 5c). Differential expression analysis of the genes involved in zebrafish heart regeneration (Hu et al. 2022) revealed that *COL12A1* was expressed in all the subpopulations of human FB, while FB4 and FB7 expressed *POSTN* and *DKK3,* respectively (Fig. 5d). All these genes were expressed by a very low percentage of cells and had an expression level lower in the human dataset than the regenerating zebrafish FB (Fig. 5d). Taken together, our data indicate that the human heart in contrast to the zebrafish does not seem to contain FB with a regenerative potential.

## Discussion

Recent years have brought an enormous development of new single-cell technologies, enabling the discovery of subtle differences in species-specific molecular programs or the relative proportions of specific cell types at unprecedented resolution. The ever-growing collection of single-cell transcriptomic data deepens our knowledge of how organisms or more specifically particular organs are built and how they function. Here, we focused on the heart, a complex organ that pumps blood in various organisms developed throughout evolution, from fish to mammals. The obvious structural and functional differences that exist among different species were addressed from the sc-seq point of view, using the human heart as a reference. By integrating single-cell transcriptomic data from adult human, macaque, mouse and zebrafish hearts considering the orthologous genes, i.e., those shared among these species, we aimed to find species-specific differences in molecular programs and cell type proportions.

While there were no obvious differences between humans and mice in the clustering of atrial CM, a mouse-specific cluster was observed for ventricular CM (vCM_mouse). Given that in four-chambered mammalian hearts the left ventricle pumps blood into the systemic circulation and that the resting heart rate in mice is approximately 10 times faster than in humans, we reason that these clustering differences could be attributed to the actual biological/functional differences (Wessels and Sedmera 2003). We observed a slightly higher expression of *Ndufa4*, *Ndufb11 and Cox7c* in the vCM_mouse cluster, which are nuclear-encoded mitochondrial genes and were previously associated with the vCM4 cluster in the human heart, characterized by a high energetic state (Litviňuková et al. 2020). Gene ontology reactome pathway analysis of the markers expressed in this cluster revealed that most of them participate in cardiac conduction (e.g., *Myh6, Pln, Kcnd2, Fgf13*). Furthermore, a mouse-specific gene *Prune2* was highly expressed in this cluster and besides showing heart-specific expression, this gene has been associated with energy metabolism in the mouse heart (Song et al. 2013). Interestingly, we also observed a low expression of *Myh7* in the mouse heart, a sarcomere gene specifically expressed in human ventricular CM. In rodents, expression of this gene is ventricular-specific during embryogenesis, but downregulated postnatally, so that in the adult mouse heart *Myh6* is the main myosin heavy chain gene, expressed both in the atria and ventricles (England and Loughna 2013). Mouse-specific cells (vCM_mouse) also expressed higher levels of *Pcdh7,* calcium-dependent adhesion molecule, as well as *Dlc1*, a Rho GTPase activating protein with tumor suppressor function that is essential for embryonic development (Durkin et al. 2005). Another striking difference between the two species is the expression of *PLCL1*, which is present in all vCM subpopulations of the human heart, but not in the mouse-specific cluster. This gene encodes a protein involved in inflammation, and an intronic variant has been associated with myocardial infarction (Lin et al. 2015; Hahn et al. 2020). Of note, cardiomyocyte clusters contain only human and mouse integration data, as the macaque dataset includes sequencing data of aortas and coronary arteries only.

Integration of PC and SMC was performed on datasets from all three mammalian species and our results show that the macaque-specific cluster (SMC_macaque) separates from the others. This cluster shows high expression of genes specific to the human SMC2 cluster (*MYH11, ACTA2, TAGLN, CNN1*), as well as *CSPG4*, a pericyte marker found also in SMCs (Murfee et al. 2005). Furthermore, this cluster expresses SMC genes specific to coronary arteries (*DES, ACTC1, SORBS2*) and the aortic arch (*POSTN*) in macaque, as described by Zhang et al., which might partially explain the separate clustering. Of note, the SMC_macaque cluster contains numerous ribosomal and mitochondrial genes, but even upon regressing them the cluster stands out.

Endothelial cells integrated well for all three mammalian species and we have not observed any species-specific clusters. However, clusters EC7_atria and EC8_ln consisted mainly of macaque and mouse cells (even though they were annotated based on the human data). In addition to the cluster-specific genes (*SMOC1, INHBA, NPR3*), EC7_atria cells were also found to express *IL13RA2* and *WIF1*, genes previously described to be specific for *M. fascicularis* EC originating from the aortic arch. In addition to the cluster-specific genes (*PROX1, TBX1, PDPN*), EC8 lymphatic cells showed high expression of *RELN*, in accordance with its specificity for the lymphatic ECs in *M. fascicularis* (Zhang et al. 2020). We have also observed that one of the marker genes for venous EC6_ven human cells—*ACKR1*—was characterized as a coronary artery-specific EC gene in the macaque dataset. Although our results show distinctively higher expression of this gene in the EC6_ven cluster as compared to other EC clusters, revisiting the single molecule fluorescent in situ hybridization (smFISH) image from the original publication showed its weak expression in arteries as well (Litviňuková et al. 2020).

Human and mouse FB integrated well, while macaque cells clumped together with the human FB3 cluster probably due to the sampling bias. The FB3 subtype of human FB was characterized by expression of the cytokine receptor genes (e.g., *OSMR, IL6ST*) and it was reported to be less abundant in the left ventricle as compared to other human fibroblast subtypes (Litviňuková et al. 2020). Consistent with this, the macaque heart dataset contains adventitial FB that make up the outermost layer of a blood vessel, composed mainly of collagen and elastic fibers secreted by FB.

Because of the evolutionary proximity, the three mammalian heart datasets integrated well, whereas the integration of the zebrafish heart dataset was challenging due to a low number of orthologous genes with the other three species, resulting in poor clustering. Therefore, we focused only on the heart FB, since they were recently shown to play a role in the heart regeneration process following injury in zebrafish (Hu et al. 2022). The regeneration-specific cell states in zebrafish (e.g., proliferating, *nppc*-, *col11a1a*-, and *col12a1a*-expressing FB) were more distant to the human cells than other zebrafish cell types, as shown by hierarchical cluster tree analysis. Importantly, these cell states exist only upon injury, and their gene expression patterns should be viewed in light of these events. Thus, the most accurate comparison of the two species would include only the non-injured heart samples, and splitting the zebrafish dataset (into control and different timepoints post-injury) enabled a better comparison to the human dataset. Our results show that all the genes relevant for regeneration in the zebrafish heart are weakly expressed in the non-injured human heart. Striking differences among the two species include expression levels of *POSTN*, *TGFB3, SFRP1,* and *DKK3*, all higher expressed in zebrafish. All these genes encode proteins that belong to TGFβ and WNT signaling pathways, which mutually interact and play key roles in fibrotic response (Akhmetshina et al. 2012).

Our study also has limitations: first, the heart tissue was sampled quite differently between mouse, human, and zebrafish compared to the macaque. Since other datasets do not sample aorta, the differences seen in the EC could have arised due to this cell population bias. Due to this sampling bias towards aorta in the macaque, comparisons with other cell types should be interpreted with caution. Second, there are strong differences in cell numbers between the species. Third, no validation experiments were performed due to very limited sample access.

In summary, we have integrated single-cell transcriptomic heart datasets of four species, and while it was straightforward for those that are evolutionarily close, divergent species could also be compared by focusing on a selected cell type. The observed species-specific differences could be explained by taking into consideration multiple factors such as functional differences, origin of tissue, and gene nomenclature.

## Supplementary Information

Below is the link to the electronic supplementary material.

Supplementary file1 (TIF 13194 KB) Integration of single cell heart transcriptome data a) Dot plot of the cell type-specific marker genes, split across species. b) 2D UMAP embedding of the integration of the cells from the three species (human dataset downsampled to the size of mouse dataset).

Supplementary file2 (TIF 4047 KB) Fibroblasts and atrial cardiomyocytes a) 2D UMAP embedding of the cell subpopulations of fibroblasts after integrating the cells from the three species. b) 2D UMAP embedding of the integration of the cells from the three species in fibroblasts. c) 2D UMAP embedding of the cell subpopulations of atrial cardiomyocytes after integrating the cells from the three species. d) 2D UMAP embedding of the integration of the cells from the three species in atrial cardiomyocytes.

Supplementary file3 (TIF 7918 KB) Gene Ontology analysis of ventricular cardiomyocytes specific to mice.

Supplementary file4 (TIF 2678 KB) Integration of cardiac fibroblasts from human and zebrafish datasets a) 2D UMAP embedding of the after integrating the cells from human and zebrafish dataset. b) 2D UMAP embedding showing the new population of fibroblasts 3 days post injury.
