# Supplementary figures and images for: Comparative single-cell analysis of the adult heart and coronary vasculature
Source: Mamm Genome. 2022 Nov 19;34(2):276–84. doi: 10.1007/s00335-022-09968-7 (PMC10290597; doi:10.1007/s00335-022-09968-7)

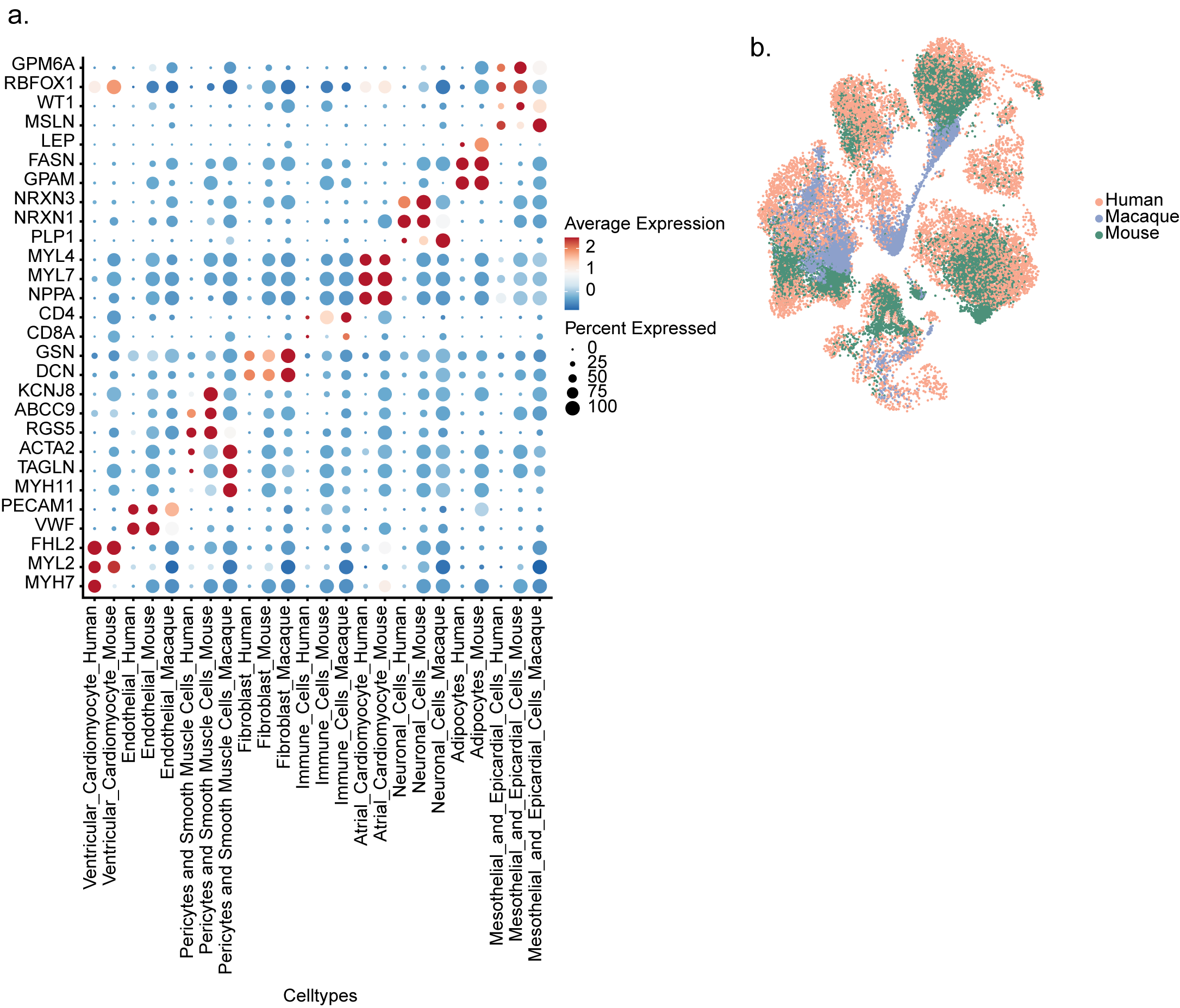

Supplement: Supplementary file 1 — Supplementary file1 (TIF 13194 KB) Integration of single cell heart transcriptome data a) Dot plot of the cell type-specific marker genes, split across species. b) 2D UMAP embedding of the integration of the cells from the three species (human dataset downsampled to the size of mouse dataset). [file 335_2022_9968_MOESM1_ESM.tif]

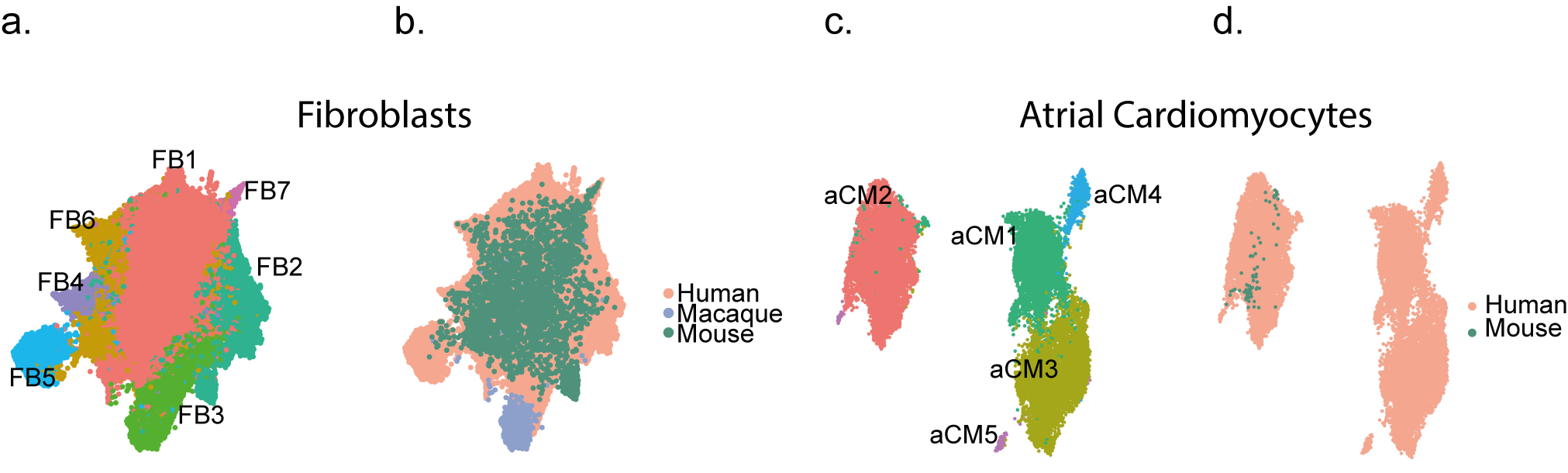

Supplement: Supplementary file 2 — Supplementary file2 (TIF 4047 KB) Fibroblasts and atrial cardiomyocytes a) 2D UMAP embedding of the cell subpopulations of fibroblasts after integrating the cells from the three species. b) 2D UMAP embedding of the integration of the cells from the three species in fibroblasts. c) 2D UMAP embedding of the cell subpopulations of atrial cardiomyocytes after integrating the cells from the three species. d) 2D UMAP embedding of the integration of the cells from the three species in atrial cardiomyocytes. [file 335_2022_9968_MOESM2_ESM.tif]

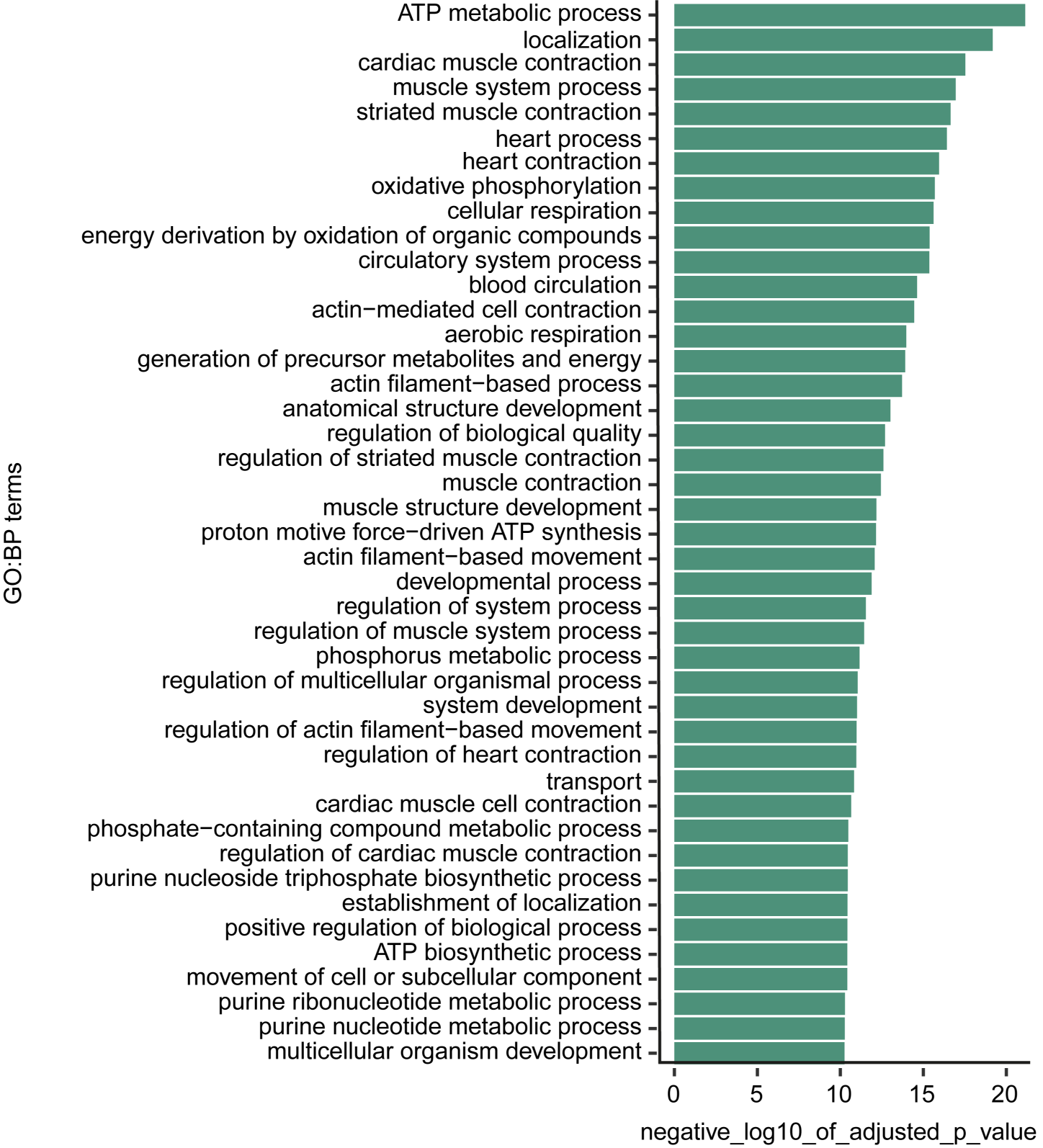

Supplement: Supplementary file 3 — Supplementary file3 (TIF 7918 KB) Gene Ontology analysis of ventricular cardiomyocytes specific to mice. [file 335_2022_9968_MOESM3_ESM.tif]

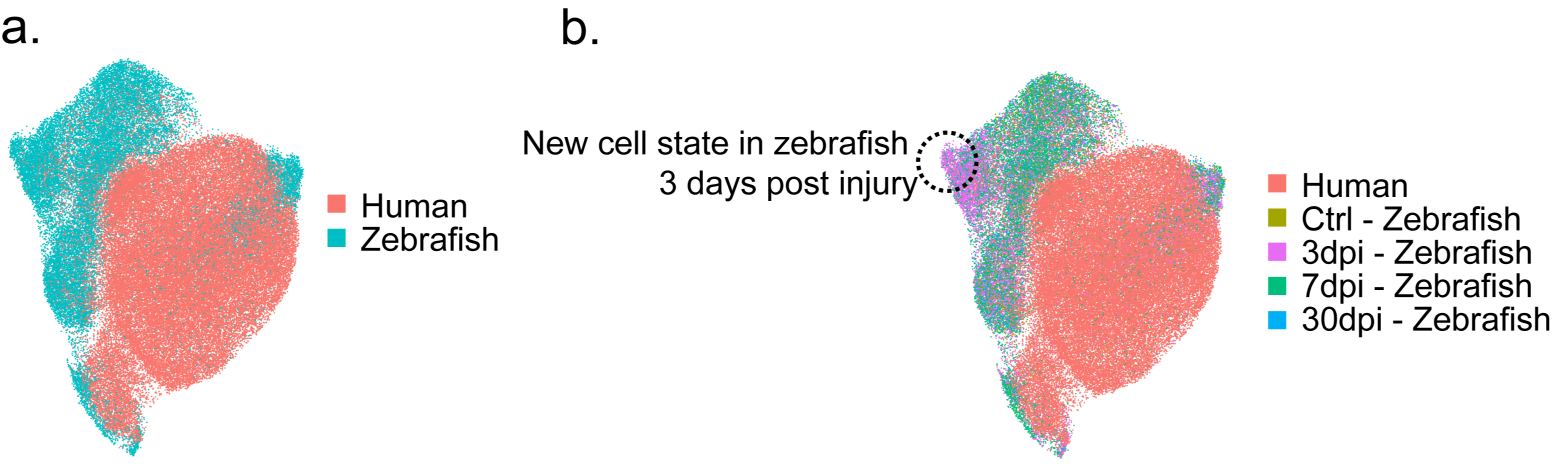

Supplement: Supplementary file 4 — Supplementary file4 (TIF 2678 KB) Integration of cardiac fibroblasts from human and zebrafish datasets a) 2D UMAP embedding of the after integrating the cells from human and zebrafish dataset. b) 2D UMAP embedding showing the new population of fibroblasts 3 days post injury. [file 335_2022_9968_MOESM4_ESM.tif]
